# Racial disparity in prostate cancer in the African American population with actionable ideas and novel immunotherapies

**DOI:** 10.1002/cnr2.1340

**Published:** 2021-02-17

**Authors:** Zachary S. Dovey, Sujit S. Nair, Dimple Chakravarty, Ashutosh K. Tewari

**Affiliations:** ^1^ The Department of Urology Icahn School of Medicine at Mount Sinai New York New York USA

**Keywords:** actionable ideas, biomarkers, genomic differences, immunotherapy, molecular differences, racial disparity, socioeconomic issues

## Abstract

**Background:**

African Americans (AAs) in the United States are known to have a higher incidence and mortality for Prostate Cancer (PCa). The drivers of this epidemiological disparity are multifactorial, including socioeconomic factors leading to lifestyle and dietary issues, healthcare access problems, and potentially tumor biology.

**Recent findings:**

Although recent evidence suggests once access is equal, AA men have equal outcomes to Caucasian American (CA) men, differences in PCa incidence remain, and there is much to do to reverse disparities in mortality across the USA. A deeper understanding of these issues, both at the clinical and molecular level, can facilitate improved outcomes in the AA population. This review first discusses PCa oncogenesis in the context of its diverse hallmarks before benchmarking key molecular and genomic differences for PCa in AA men that have emerged in the recent literature. Studies have emphasized the importance of tumor microenvironment that contributes to both the unequal cancer burden and differences in clinical outcome between the races. Management of comorbidities like obesity, hypertension, and diabetes will provide an essential means of reducing prostate cancer incidence in AA men. Although requiring further AA specific research, several new treatment strategies such as immune checkpoint inhibitors used in combination PARP inhibitors and other emerging vaccines, including Sipuleucel‐T, have demonstrated some proven efficacy.

**Conclusion:**

Genomic profiling to integrate clinical and genomic data for diagnosis, prognosis, and treatment will allow physicians to plan a “Precision Medicine” approach to AA men. There is a pressing need for further research for risk stratification, which may allow early identification of AA men with higher risk disease based on their unique clinical, genomic, and immunological profiles, which can then be mapped to appropriate clinical trials. Treatment options are outlined, with a concise description of recent work in AA specific populations, detailing several targeted therapies, including immunotherapy. Also, a summary of current clinical trials involving AA men is presented, and it is important that policies are adopted to ensure that AA men are actively recruited. Although it is encouraging that many of these explore the lifestyle and educational initiatives and therapeutic interventions, there is much still work to be done to reduce incidence and mortality in AA men and equalize current racial disparities.

## 1 INTRODUCTION

African Americans (AAs) in the United States have higher incidences and mortality for a number of cancers.^1^ Their lifetime risk of prostate cancer (PCa) is 1 in 6, with a lifetime risk of PCa specific mortality of 1 in 23.^1^ For non‐Hispanic Whites their risk of PCa is lower with incidence of 1 in 8, and mortality of 1 in 42.^1^ The drivers of this epidemiological disparity in incidence and mortality are not clearly defined and complex, ranging from social networks also causing lifestyle and dietary issues, healthcare access problems, comorbidities, and their influences on pelvic inflammation and tumor microenvironment, as well as tumor biology with associated differences in genomic, molecular, and immunological pathways.^1^, ^2^, ^3^ Which of these drivers is the most significant has been debated in the literature for some time, but, although there are clearly differences in tumor biology, more recent reports suggest once socioeconomic differences are removed, prostate cancer specific mortality rates for African and Caucasian men are similar.^4^, ^5^, ^6^ Examining retrospective data in PCa patient cohorts from the Veterans Affairs health care system, as well as from Surveillance, Epidemiology, and End Results (SEER) database and four Radiation Therapy Oncology Group trials with long‐term follow‐up, showed once AA men have equal access to healthcare and standardized treatment, prostate cancer specific mortality (PCSM) is similar for AA and CA men.^4^, ^5^ Similarly, Tewari et al^6^ in a PCa patient cohort from the Henry Ford Health Care System found differences in PCSM no longer existed after multivariate analysis adjustment for socioeconomic status. Nevertheless, the higher incidence of PCa in AA men remains, and given their reduced access, one would expect the reverse. Clearly, any discussion on racial disparity with a view to producing equity in PCa incidence and management outcomes needs to be multifactorial and is complicated by the fact that “race” is a social construct rather than a biological definition. AA men have diverse and genetically heterogeneous ancestry, not only within Africa but also Europe and the Americas, and so their molecular and immunological response to cancer as well as their tumor biology is likely to be equally diverse.^7^

In addition to this recent epidemiological evidence, there have been reviews of the molecular and genomic aberrations in African American (AA) men, and detailed analyses of the molecular and genomic pathways of PCa for all ethnicities at times highlighting potential therapeutic targets.^8^, ^9^, ^10^, ^11^ Bhardwaj et al^8^ outlined the molecular differences in PCa between AA and CA men, discussing genetic polymorphisms, gene mutations, epigenetic changes, and microRNAs, as well as comparative aberrations in the androgen receptor (AR), growth factor receptor (GFR), and inflammatory signaling pathways. For all ethnicities, an understanding of prostate cancer oncogenesis has evolved significantly, with emerging research using this body of knowledge to explore novel immunotherapeutics and plan “precision” treatments, aimed specifically at an individual's unique disease. Mitchell and Neal^10^ elegantly describe the genomic evolution and mutational pathways of PCa, with ETS fusion positive and negative tumors, as well as the influence on oncogenesis of AR signaling and developmental pathways. Wang et al^12^ use a comprehensive review of prostate cancer genetics listing 33 of the commonest genetic alterations with their biological function to highlight emerging targeted therapies and immunotherapies, including apalutamide (an AR antagonist), atrasentan (antagonist of the endothelin receptor), and denosumab (RANKL targeting monoclonal antibody) as well as PCa vaccines and checkpoint inhibitors, respectively. Moreover, all forms of immunotherapy are a potentially exciting and efficacious modality of treatment for localized and metastatic prostate cancer, including vaccines, checkpoint inhibitors, adoptive cell therapy, antibody therapy, and radioimmunotherapy, although there have been few studies focused specifically on immunotherapy for the AA race.^13^ Interesting studies on noncoding and micro‐RNAs have shown differential expression in AA men as compared to CA men,^14^ and with specific reference to the tumor microenvironment (TME), Gillard et al^15^ have shown a more reactive stroma with enhanced chronic inflammatory infiltrate as well as fibroblast function in AA men.

As yet, to our knowledge, there have been no reports leveraging our understanding of molecular differences in PCa in AA men into clinical practice. This review will first frame PCa oncogenesis across all ethnicities in Hannahan and Weinberg's “Hallmarks of Cancer”,^16^ which is presented concisely in Table 1. This table aims to be used as a reference to benchmark molecular and genomic differences in AA men that have emerged in the recent literature, including differences in the tumor microenvironment and noncoding RNA. Once highlighted, and bearing the recent epidemiological evidence in mind, these key insights will be used as a stepping stone to devising actionable ideas and novel immunotherapies with the ultimate aim of providing at least some guidelines for reducing the incidence and mortality of PCa in the United States in African American men.

**TABLE 1:** Summary of prostate cancer oncogenic pathways

Hallmark	Pathways, Gene or Mechanism	Description	Function	Clinical aspects	References
Constitutive proliferation	P13‐AKT pathway	Family of enzymes forming part of the PI3K‐AKT mammalian target of rapamycin pathway (mTOR) pathway.	Membrane receptor tyrosine kinases mediating intracellular serine and/or threonine phosphorylation of a range of downstream substrates, to produce cell proliferation, motility, and survival.	Activation of PI3K by PTEN loss is thought to occur at the initial stages of prostate oncogenesis, with further mutations such as ETS fusion hallmarking the progression to more invasive disease. The combination of PTEN loss, with additional deletions or mutations of RB1 and TP53 have also been shown to harbor the onset metastatic castration resistant PCa (mCRPC) phenotypes in both human samples and genetically engineered mouse models.	10, 11, 17, 18, 19
	MAPK/ERK pathway	G protein coupled membrane tyrosine kinase receptor.	Downstream intracellular activation of nuclear transcription factors eg, c‐myc via Ras, Raf, and ERK proteins, regulating cellular differentiation and proliferation.	There have been reports of aberrant MAPK/ERK function in advanced prostate cancer with enhanced AR signaling, but overall its role in prostate cancer requires further study.	20, 21
	Androgen receptor (AR) pathway	Cytoplasmic androgen receptor consisting of N‐terminal, DNA, and ligand‐binding domains.	Aberrant AR signaling is, the driving force for metastatic and castration resistant disease, with 60% of ARs abnormal in CRPC. Mutations in AR transcriptional cofactors and regulators may have influence, such as NCOA2 which is enhanced in 8% of primary and 37% of metastatic disease cases.	Potential mechanisms of AR activation despite lack of androgens include mutations of the AR receptor itself, posttranslational modification of the AR receptor, mutations in transcriptional co‐regulators (eg, NCOA and NCOR co‐regulator groups), androgen synthesis within the tumor, activation of AR modulatory kinase pathways resulting in hormone dependent but androgen independent activity (eg, via PI3K activation) and altered AR degradation (eg, by E3 ubiquitin ligases Mdm2 activity. AR splicing variants (ARVs) have been proposed as another mechanism for castration resistance, as well as resistance to enzalutamide and abiraterone.	11, 22, 21, 23, 24
	C‐MYC (gene locus 8q24.21)	Transcription factor from the basic helix‐loop‐helix leucine zipper (bHLHZ) family.	Promotes oncogenesis through activation protumorigenic factors influencing cell cycle progression and survival.	Enhanced activity has been demonstrated in 8% of localized PCa and 30% of cases with advanced disease, correlating with higher Gleason grade and poor survival.	25, 26, 27, 28, 29
Uncontrolled cell division	P53 (gene locus 17p13.1)	Tumor phosphoprotein 53	Tumor suppressor that acts to prevent cells from entering the S phase of the cell cycle, as well as promoting apoptosis in cells with abnormal DNA.	P53 mutations are more common in advanced, metastatic and castration resistant disease than localized disease (over 40% compared with less than 20% respectively). The combination of P53, PTEN, and RB loss is associated with progression to mCRPC and specifically the neuroendocrine phenotype, which is indifferent to AR signaling.	25, 19, 30, 31, 32, 33
	RB (gene locus 13q14.2)	Retinoblastoma protein	Controls progression from the G1 to S cell cycle phase. Loss of RB proteins lead to E2F activity, resulting in upregulation of genes promoting G1/S transition and cell cycle progression.	Mutations or deletions are more common in castration‐resistant rather than localized PCa with up to 45% of mCRPC patients affected. The timing of mutation may suggest the development of castrate‐resistant disease.	10, 34, 19, 21, 35
	PTEN (gene locus10q23.31)	Phosphatase and Tensin homologue protein, a phospholipid phosphatase	Indirectly inhibits the phosphatidylinositol 3‐kinase‐protein kinase B (PKB‐Akt) signaling pathway, which itself plays a key role in the cell cycle. PTEN also effects the levels of tumor suppressor gene CDKN1B (coding the p27 protein), which also plays a crucial role in cell cycle control at the G1/S transition phase.	A recent randomized Phase II study showed AKT inhibition with ipatasertib in combination with abiraterone showed improved anti‐tumor activity measured by radiographic progression‐free survival in mCRPCa patients, especially those with loss of PTEN signaling.	25,26, 36, 37, 38, 39, 40
	PHLPP1 (gene locus 18q21.33)	PH domain and Leucine‐Rich Repeat *Protein* Phosphatase 1	Tumor suppressor gene coding for a phosphatase that inactivates the PI3‐AKT pathway.	Induces PCa with associated PTEN in knockout mice. Co‐deletion of PHLPP1 and PTEN is closely correlated with metastatic disease. interestingly, low *PTEN/PHLPP1* transcription also correlates with biochemical relapse in patients after prostate surgery.	18
Overriding cell death	FOXA1 (gene locus 14q21.1)	Androgen receptor cofactor	It functions to promote Androgen induced cellular proliferation and epithelial cell differentiation. A pro‐apoptotic role via alteration of bcl‐2 expression has been shown, suggesting mutations in FOXA1 may contribute to PCa cells avoiding cell destruction.	Common in castration resistant disease where its protein promotes cell cycle progression.	12, 34
Loss of cell cycle control	TERF1 (gene locus 8q21.11)	Telomere specific protein forming part of the shelterin nucleoprotein complex.	Acts by inhibiting telomerase and limiting the elongation of individual chromosome ends. Mutations in this gene allow activation of telomerase that contributes to unlimited replicative capacity.	Specific role in prostate cancer oncogenesis and metastatic progression requires further study.	41
	TNKS (gene locus 8p23.1)	Tankyrases (TNKS) are multifunctional poly(ADP‐ribose) polymerases catalyzing ADP‐ribosylation.	The enzymes target proteins are involved in WNT signaling, telomere maintenance, and mitosis regulation. They also regulate tumor suppressors, including AXIN, phosphatase and tensin homolog and angiomotin.	Tankyrase inhibitors may exert a therapeutic effect in prostate cancer by inhibiting telomerase activity and Wnt signaling, mediated by TRF1 and AXIN stabilization respectively. Studies have demonstrated the C44 molecule (a putative tankyrase inhibitor) was able to reduce PCa cell proliferation in vitro and in vivo.	42, 43
	SHQ1 (gene locus 3p13‐14)		SHQ1 is part of a gene locus including FOXP1 that is commonly deleted in PCa. In association with PTEN loss, FOXP1‐SHQ1 deletion causes cellular growth and anaplasia, as well as stimulating mTORC1 and reducing Akt phosphorylation	FOXP1‐SHQ1 loss combined with PTEN loss is linked to biochemical recurrence after primary treatment for localized PCa.	44
Tumor‐induced angiogenesis	HOX transcription factors	Includes HOXB13 and HOXB7 proteins.	Supports tumor growth and microenvironment by promoting angiogenesis through the secretion of proangiogenic cytokines.	HOXB13 has been extensively investigated as a hereditary susceptibility gene if mutated, especially the G84E subtype, found in 5% of families with inherited disease. HOXB13 protein has a number of downstream protumorigenic effects including, eg, repressing p21 tumor suppressor gene, and upregulation of zinc transporters, so reducing intracellular zinc levels, reducing the level of inhibitor of NF‐κB alpha (IκBα), which then promotes disease progression. HOXB7, which is overexpressed PCa, has been shown to induce tumor cell secretion of FGF2 vascular endothelial growth factor A (VEGFA), C‐X‐C motif ligand 1 (CXCL1), and interleukin 8.	45, 46
	NF‐kB (gene locus 4q24)	NF‐kB family of transcription factors	Gene regulators linked to inflammatory processes (see below) but may also be involved in angiogenesis.	Interaction of NF‐kB with the PKC pathway and related signals, including c‐Rel transcription factor, upregulates transcripts promoting angiogenesis, but the details of this interaction require further study.	47
Tumor Invasion and metastatic cascade	EGFR (gene locus 7p11.2)	EGFR is a transmembrane protein and member of the ERB family of receptors.	Intracellular signaling cascades include PI3K‐AKT and MAPK/ERK pathway leading to cellular proliferation, inhibition of apoptosis, angiogenesis, migration, adhesion, and invasion.	Long been recognized as associated with recurrent disease and progression to castration resistance in PCa. EGFR activity promotes cellular invasion by regulating urokinase‐type plasminogen activator activity. promotes survival of initiating and circulating tumor cells that metastasize to bone, whereas HER2 supports cellular growth once they have reached their metastatic sites.	20, 48, 49
	RAS (gene locus 11p15)	RAS protein is a guanosine‐nucleotide binding protein.	RAS protein, when bound to GTP, has high affinity for a number of effectors including RAF protein in the MAPK/ERK pathway, and PI3K.	Loss of the RAS GTPase‐activating protein (RasGAP) and the gene *DAB2IP* induced metastatic PCa in a mouse model. Notably, DAB2IP functioned as a signaling scaffold coordinating tumor growth and metastasis by RAS and NF‐κB respectively. *DAB2IP* is suppressed in human PCa and epigenetic suppression of *DAB2IP* is an important means by which the polycomb‐group protein histone‐lysine *N*‐methyltransferase EZH2 stimulates RAS and NF‐κB to promote metastasis.	20, 50
	RKIP (gene locus 12q24.23)	Raf kinase inhibitor protein (RKIP) is a phosphatidylethanolamine binding compound.	Inhibit the MAPK/ERK pathway by binding to RAS 1 protein.	Suggested as a metastasis suppressor gene on the basis of reduced expression in localized PCa cell lines and its metastatic derivatives. Promotes apoptosis, so reduced RKIP expression protects cancer cells against cell death, and restored expression in metastatic cells inhibits progression.	51, 52, 53
	RalGEF/Ral pathway	RaLGEF/Ral pathway is a downstream effector pathway of the Ras protein discussed above.	Similar signaling functions as RAS 1 protein, with inhibition of MAPK/ERK pathway.	Using assays from a non‐metastatic human PCa cell line, studies have shown activation of the Ral guanine nucleotide exchange factors (RalGEFs) pathway led to bone metastases. RalA loss in metastatic PC3 cells inhibited progression to bone metastasis and suppressed further growth of metastasis in bone, whereas “homing and initial colonization were less affected”.	54
	SPINK1	Serine peptidase inhibitor Kazal type 1 (SPINK1) is a serine protease inhibitor	SPINK1 has shared homology with EGF, and so may stimulate growth factor signaling pathways.	Associated with an aggressive subset of ETS negative PCa. SPINK1 overactivity resulted in progression and cancer spread via epidermal growth factor receptor in experimental mouse models. Studies show positive correlation between SPINK1 overactivity and progression to metastasis after PSA recurrence.	55, 56, 57
Genetic variations and chromosomal instability	CHD1 (gene locus 5q15)	Chromodomain helicase DNA‐binding protein 1 gene.	Enzyme involved in chromatin remodeling and the function of maintaining the configuration of chromatin throughout the genome	CHD1 loss Linked to ETS fusion‐negative tumors. It occurs in 10% to 25% of ETS negative PCa. It's loss results in significantly more chromosomal rearrangements or “chromoplexy” (up to seven times more).	10, 12, 34, 58
	SPOP (gene locus 17q21.33)	Speckle‐type POZ protein (SPOP) gene on the q arm of chromosome 17	SPOP mutations lead to impaired DNA repair by causing elevated replication stress, with reduced repair of stalling from induced replication forks prone to deletions and mutations associated with ETS fusion‐negative tumors	Prone to deletions and mutations associated with ETS fusion‐negative tumors. SPOP functions as an E3 ubiquitin ligase substrate‐binding protein and is lost in up to 15% of tumors overall. Deletion of SPOP is also thought to be oncogenic by stabilizing the DEK oncogene due to loss of ubiquitin ligase activity, which itself promotes PCa epithelial cell invasion in PCa cell lines.	10, 34, 58, 59
	NKX3.1 (gene locus 8p21.2)	Homeobox transcription factor NKX3.1 is associated with both ETS negative and positive tumors and has been proposed as a tumor suppressor.	NKX3.1 complexes with AR to facilitate DNA repair of abnormal DNA generated during transcription, and also activates the ATM gene (see below).	It has been shown to play a critical role in stem cell function and the protection of prostate epithelial cell DNA damage in the mouse. PSA regulated physiologically, but also epigenetic modification is thought to play a key role in its downregulation. Downregulated in early PCa and may act as “gatekeeper” in PCa initiation, as well cellular proliferation and invasion.	12, 60, 61
	ATM (gene locus 11q22.3)	PI3K‐related serine/threonine protein kinase (PIKK).	Key role in DNA double‐strand break repair (DSB).	Germline ATM mutations been previously suggested as a risk factor for PCa. In association with other DNA repair genes (BRCA1 and BRCA2), a risk factor for distinguishing indolent from lethal disease.	62, 63, 64
	EZH2 (gene locus 7q36.1)	Enzyme histone methyltransferase EZH2 (Enhancer of zeste homolog 2).	Functions to keep the epigenomics of the cell healthy, so maintaining the integrity of tumor and metastasis suppressor genes for PCa.	Examining gene expression profiles in PCa patients, it was found to be upregulated in advanced PCa, and due to its interaction with AR‐signaling pathways as coactivator, associated with advanced disease progression.	65, 66
	DNA Methylation	Commonest epigenetic change inducing either gene suppression or expression	One of many epigenetic changes implicated in PCa development and progression.	Common examples including GSTP1 gene promoter with loss of expression, present at all stages of PCa and the HOX gene family loci, which also affected in a recurrent fashion at different stages of the disease.	58, 67, 68, 69, 70, 71, 72
Pro‐tumor inflammation	Interleukin‐6	Pro‐inflammatory Cytokine expressed by prostate cancer cells and tumor stromal environment.	Acting by the Janus kinase and STAT3 signaling pathways. Mesenchymal stem cells and stromal cells have upregulation of IL‐6 expression, which supports the paracrine IL‐6 signaling function in PCa.	It is thought to be involved in the development of castration resistance metastasis (although clinically antibody to IL6 was unsuccessful as a therapeutic option for this patient group), the regulation of cellular stemness by STAT3 phosphorylation, resistance to the non‐steroidal anti‐androgen enzalutamide, as well as demonstrating anti‐apoptotic effects and so potentiating PCa cellular survival.	73, 74
	NF‐kB (gene locus 4q24	NF‐κB transcription factors proteins	Gene regulators linked to inflammatory and immune activation, cell proliferation and anti‐apoptotic effects exerting control of DNA transcription and inflammatory cytokine production	NF‐κB promotes cell survival and invasion, metastatic progression and loss of androgen receptor expression and castration resistance, shown in PCa cell lines and mouse models. The fact that NF‐kB signaling is activated by inflammatory cytokines within the tumor environment, has led to the suggestion it may be a key link between inflammation and cancer, and potentially a target for therapeutic intervention.	75, 76, 77
Metabolic reprogramming	NT5E (CD73) (gene locus 6q14.3)	Ecto‐5′‐nucleotidase present on various cell surfaces,^78^ that lyses extracellular adenosine monophosphate into adenosine and inorganic phosphate.	It lyses extracellular adenosine monophosphate into adenosine and inorganic phosphate. Since the free adenosine inhibits cellular immune responses, it acts as an inhibitory immune checkpoint agent, promoting immune escape of tumor cells	NT5E contributes to cancer development and sustaining angiogenesis in PCa murine tumor models. NT5E inhibition has been shown to reverse PCa immune escape and cause in vitro destruction of cancer cells by cytotoxic CD8^+^ T cells and NK cells, but not specific to PCa.	78
Escaping anti‐tumor immune response	Immune escape mechanisms			Reduced sensitivity to the inhibitory effects of TGF‐Beta. Enhanced tumoral WNT signaling resulting in immune suppression, which is exacerbated by T regs and mesenchymal derived stem cells in the TME. Impaired antigenicity by dendritic cells with loss of MHC1.	79, 80
	BTNL2 (6p21.32) gene	Negative T‐cell immune surveillance factor	Reducing T cell proliferation and cytokine production	Although rare, BTNL2 mutation has been shown to increase the risk of both hereditary and sporadic PCa. Interestingly, it has also been implicated in the etiology of inflammatory diseases such as sarcoidosis and ulcerative colitis.	81
Tumor Microenvironment	PCa stromal tissue	Contains endothelial cells, nerve cells, and fibroblasts with also non cellular elements (enzymes, matrix, and growth factors)	In tumor states, this provides a framework facilitating oncogenesis and disease progression Also results in Epithelial to Mesenchymal Transition (EMT), a program of events that include stromal cells developing an altered phenotype more resembling myofibroblasts expressing vimentin and smooth muscle actin, similar to the process of wound healing	In keeping with the process of wound healing, the TME and process of EMT promotes increase protease activity, angiogenesis and inflammatory cells, but rather than laying down granulation tissue they cause stromal reactivity and tumor cell proliferation. A recent interesting study examined EMT in a mouse model focused on bone marrow dissemination . They confirmed ex vivo tumor cells showed increased angiogenic, proliferative and migratory features with altered mesenchymal markers including E‐cadherin, Snail, ZO‐1, and vimentin.	15, 82, 83, 84, 85

## 2 GENOMIC AND MOLECULAR FUNDAMENTALS IN THE ONCOGENESIS AND PROGRESSION OF PROSTATE CANCER

**FIGURE 1:**
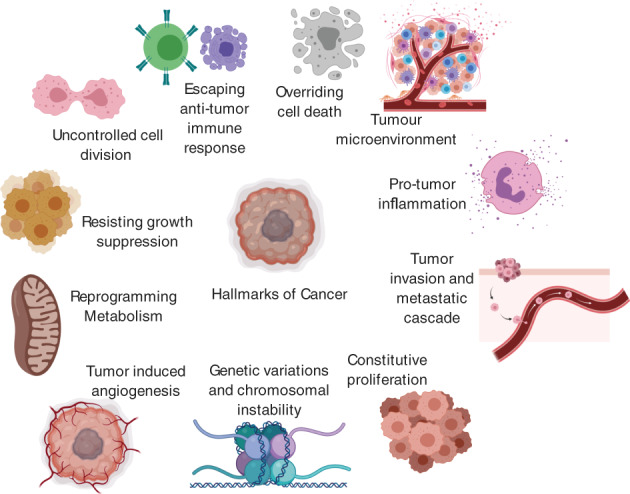
The Hallmarks of Cancer outlining a synopsis of oncogenesis applicable to different cancers and ethnicities. Adapted from reference 16 with permission from Elsevier

In their original paper, Hanahan and Weinberg^86^ outline 6 “Hallmarks of Cancer” as an organizing principle to explain oncogenesis and its progression. This included constitutive proliferation, uncontrolled growth, loss of cell cycle control, overriding cell death, tumor‐induced angiogenesis, and tumor Invasion and metastatic cascade.” Later, they acknowledged the importance of “genetic instability, inflammation, metabolic reprogramming, escaping antitumor immune response, and tumor microenvironment” (see Figure 1 and Table 1). The genomic and molecular pathways to the development and progression of PCa have also been well described,^10^, ^11^, ^25^ and this knowledge will be reframed across all ethnicities under Hanahan and Weinberg's Hallmarks.^16^ Each of the Hallmarks' genetic or pathway abnormalities have been outlined concisely, with specific reference to prostate cancer in Table 1. This Table has been presented for use as a reference, to highlight the discussion of genomic and molecular differences in the AA population. At the time of writing, not all these pathways have demonstrated differences in the AA population, and so may not contribute to a coherent story of PCa tumor biology in AA men. Nevertheless, they have been included, not only for completeness, but also as a reference should more differences emerge in the literature and potentially to stimulate further research for this important topic. There is also an additional section on putative genetic subclasses of PCa.

### 2.1 Putative disease subclasses

ETS FUSION POSITIVE TUMOURS: E26 Transformation Specific (ETS) transcription factor gene fusions are found in approximately half of prostate cancers,^87^ the commonest of which is fusion of the androgen‐responsive promoter Transmembrane Protease Serine 2 (TMPRSS2) with the ERG gene of the ETS family.^55^ Other less common ETS transcription factor gene fusions include Friend leukemia virus integration 1 (FLI1) and ETS variants 1, 4, and 5 (ETV1, ETV4, and ETV5).^10^, ^88^ This has led to a genetic based disease classification of PCa into ETS fusion positive and ETS fusion negative tumors. The fusion is thought to occur early in oncogenesis, being present in prostatic intraepithelial dysplasia (PIN), and causes altered expression of other genes implicated in oncogenesis, such as MYC, NKX3.1, EZH2, and SOX‐9.^89^, ^90^, ^91^ Once ETS Fusion has occurred, disease progression is likely to require interaction with other aberrant pathways, for example, PTEN inactivation discussed in Table 1.^87^, ^92^, ^93^, ^94^ Most likely due to tumor heterogeneity, studies of the clinical and prognostic significance of ETS fusions have not produced any consistent results.^88^ More study is required in this area.

ETS NEGATIVE TUMORS: this accounts for the other half of prostate cancers and has been putatively subclassified into three groups according to three dominant different genomic drivers. These are SPINK1 overexpression, SPOP mutation, and CHD1 deletion.^10^, ^11^ The details of these molecular pathways are summarized concisely in Table 1.

## 3 SPECIFIC FINDINGS IN AFRICAN AMERICAN MEN

**FIGURE 2:**
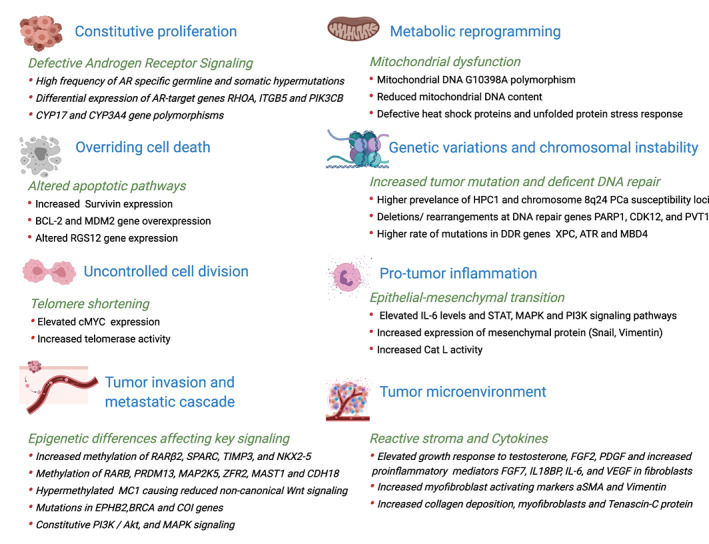
Key molecular characteristics of AA tumors with specific reference to differences in prostate cancer oncogenesis between AA and CA populations

Having outlined the fundamentals of oncogenesis for prostate cancer according to the “Hallmarks of Cancer”^16^ in Table 1, studies highlighting specific differences for AA versus CA men will be discussed under the same headings. Given that some of the studies broadly investigate differences using, for example, GWAS, there is inevitable crossover of data, and some of these Hallmarks have been discussed in combination. At the end of this section, the importance of micro and noncoding RNA disparities are addressed, as well as anatomical and molecular correlations and putative disease subclassifications. A summary of the current molecular and genomics disparities in AA men is illustrated in Figure 2.

### 3.1 Constitutive proliferation

#### 3.1.1 Androgen receptor signaling

The androgen hormonal axis, the androgen receptor (AR), and its signaling pathway all play a critical role in PCa oncogenesis and management. Mutations in the AR are uncommon in the treatment of naïve PCa but can be found in up to 15% of androgen independent disease, heralding the onset of castrate resistance, and potentially acting as a biomarker of progression.^95^ Moreover, many studies have shown clear differences in AR mutations and signaling between AA and CA populations. Where possible, the results of clinical studies have been presented, but the detailed frequencies of different AR mutations or mutations in the genes of the androgen biosynthetic pathway in AA versus CA populations require further study.

Androgen receptor (*gene locus Xq11*) and target genes: The AR's first exon contains two polymorphic nucleotide repeats, CAG and GGN,^96^ and transcriptional activity of an AR correlates inversely with CAG repeat length.^96^, ^97^ Short CAG and GGN repeat length are also linked to increased risk of PCa,^98^, ^99^, ^100^ and AA men more commonly have fewer CAG repeats than CA men.^101^, ^102^ Bennett et al^102^ examined CAG repeat length in the AR gene in 151 AA and 168 CA PCa patients, finding AA men had significantly less mean CAG repeats (19.8 vs 21.9), and men with shorter CAG repeats were more likely to have metastatic disease. The specific association of polymorphic GGN repeats with PCa is more controversial with conflicting reports of its increase and effects in ethnic populations.^96^, ^103^, ^104^, ^105^

AR derived protein expression has been investigated by immunohistochemistry in benign prostatic hyperplasia and PCa tissue.^106^ Nuclear AR expression in epithelial tissue was significantly increased in AA patients (*P* = .048).^107^ In contrast, stromal AR levels are decreased in PCa and there is a greater level of decrease in AA compared to CA patients.^108^, ^109^ The contrasting expression of epithelial and stromal AR in PCa may be one of the commonest causes of castration resistance in PCa.^96^ Higher epithelial AR levels in AA men would suggest the expression of AR target genes is also increased. Using gene expression profiling, Wang et al highlighted 1188 genes that are differentially expressed in AA men including AR‐target genes RHOA, ITGB5, and PIK3CB that are all linked to increased invasiveness of PCa cells.^96^, ^110^

AR hypermutations: unique AR specific germline and somatic hypermutations have been shown in higher frequency in AA over CA men.^111^ Examining 200 AA and 100 CA patients with sporadic primary PCa, somatic missense AR mutations were found in 8.5% and 2% in the AA and CA groups, respectively. Analysis of the same groups revealed AR germline mutations were four times more common in the AA group.^111^ One of the commonest somatic mutations, which occur in PCa AR mutation, is Thr877Ala.^112^ A recent mutation study characterized AR interacting proteins known to bind T877A‐AR, in different racial groups, and those unique to AA men included DNA damage excision repair proteins such as ERCC1, ERCC2, ERCC3, ERCC5, and FEN1.^96^, ^112^

### 3.2 Genes involved in Biosynthetic enzymes affecting androgen

*SRD5A2* (*gene locus 2p23*): Genetic mutations in 5α‐reductase (encoded by the SRD5A2 gene and catalyzing the conversion of testosterone to dihydrotestosterone (DHT)) may be altered in racial groups, for example, certain SRD5A2 polymorphic alleles, such as 121‐131‐bp allele, are restricted to AA men.^113^ DHT's affinity for the AR is five times that of testosterone, and although the significance of this enzymic alteration is not clear and macroscopically androgen testosterone levels in AA and CA men have been shown to be similar, in the context of increased AR tissue expression, alterations in DHT levels may have consequence at a cellular level.^114^

*CYP17* polymorphism (gene locus 10q24): The CYP17 gene is on chromosome 10 and codes for the cytochrome P450‐c17a enzyme in the steroid biosynthetic pathway.^115^, ^116^ T‐to‐C polymorphism in the 5' promoter region causes A1 (T) and A2 (C) alleles^117^ that may be linked to greater PCa risk.^118^, ^119^, ^120^ Although the reports are somewhat conflicting^121^, ^122^, a study that investigated CYP17 polymorphism in three different populations showed that AA men with A2 (C) allele had an increased risk for higher grade and stage PCa.^96^, ^116^

*CYP3A4* polymorphism (*gene locus 7q21*.*1*): The CYP3A4 gene is from the cytochrome p‐450 family, codes for an enzyme that oxidizes testosterone to less active metabolites.^96^, ^123^, ^124^ Race‐stratified analyses showed that a germline genetic variant CYP3A4*1B was associated with aggressive PCa in AA men.^125^ This is in keeping with other reports demonstrating this mutation is linked to worse outcomes in AA men.^126^

Serum Androgen levels: The length of androgen exposure has previously been implicated in PCa development,^96^ and young AA men have been shown to have androgen levels 15% higher than CA men,^127^ with associated increase in levels of 5‐alpha reductase.^128^, ^129^ Nevertheless, a causative relationship between prostate cancer incidence and elevated androgen levels remains controversial.

#### 3.2.1 EGFR signaling

EGFR signaling is important in PCa progression independent of AR signaling pathways,^130^ as discussed in the section above. It is increased in 40% to 80% of PCa patients and is more common in AA men. EGFR expression is associated with higher Gleason scores and castration resistant disease.^130^, ^131^, ^132^ Unfortunately, Phase‐II studies of the EGFR inhibitor gefitinib failed to demonstrate any effect on PSA levels, despite low toxicity,^131^, ^133^ and in resistant disease, it may be linked to PI3K/Akt pathway hyperactivation.^130^, ^134^, ^135^

#### 3.2.2 Uncontrolled cell division and overriding cell death

Bcl‐2 (*gene locus 18q21*.*33*): Bcl‐2 is an oncogene coding for a protein that inhibits apoptosis.^96^, ^117^, ^136^ A number of studies have shown altered apoptosis and proliferation status associated with Bcl‐2 in AA and CA men. De Vere White et al^137^ found Bcl‐2 and proliferation status was linked in AA men, but not in CA men, and Gao et al,^22^ in post‐radical prostatectomy patients, found significantly higher apoptosis levels in AA rather than CA men but similar levels of proliferation. Taking a different perspective, Khan et al^138^ looked at expression of the apoptosis inhibitor protein, survivin, in blood‐derived exosomal vesicles of AA and CA men with PCa. AA men had higher levels of survivin protein and higher levels of exosomes. Drawing any clear conclusions from these studies is challenging, suffice to say levels of apoptosis and proliferation are important to PCa aggressiveness and spread, and clearly are different in AA and CA men.

MDM2 (*gene locus 12q15*): The human mouse double‐minute 2 protein (Mdm2) is a ubiquitin ligase that supports the degradation of p53 protein, itself crucial for apoptosis, DNA repair, and cell cycle arrest.^96^ An association between altered Mdm2 expression and increased risk of localized and advanced PCa risk has been demonstrated in a number of studies.^139^, ^140^, ^141^, ^142^ Moreover, a single nucleotide polymorphism in the promoter region (SNP309) of this gene results in increased transcription and suppression of p53 activity.^96^, ^143^ Despite a large meta‐analysis by Yang et al^144^ showing an association between the SNP309 polymorphism of Mdm2 with reduced risk of PCa in CA men, there was no association with PCa in men of different ethnicity.^144^

Other studies have investigated gene alterations in AA men, which influence apoptotic pathways. Examining the PTEN status in AA men using tissue microarray on radical prostatectomy specimens, Tosoian et al^145^ found PTEN loss is significantly less in AA men than CA men, but if present, was nevertheless associated with biochemical recurrence and metastasis. Wang et al^146^ identified a putative tumor suppressor in AA men, RGS12, that influences apoptosis by reducing MNX1 and AKT levels.

#### 3.2.3 Loss of cell cycle control

Telomere shortening occurs early in prostate cancer oncogenesis, possibly due to oxidative stress and local inflammation, and it can also increase genomic mutational burden.^41^ Ongoing telomere shortening is not compatible with cancer cell survival, and in clinical and advanced prostate cancer, telomerase activity is increased to maintain replicative immortality. The key step in telomerase activation is expression of a catalytic subunit TERT, such that the activity of telomerase directly correlates with TERT expression.^41^, ^147^, ^148^ Studies have shown c‐MYC oncogene overexpression brings about TERT overexpression,^41^, ^149^, ^150^ and the MYC transcription factor is known to bind to the TERT promoter.^151^ In AA men, both c‐MYC oncogene expression and telomerase activity are elevated and at a level that is significantly higher than for CA men,^9^ so maintaining the cancer cells' replicative potential.

#### 3.2.4 Tumor Invasion and metastatic cascade

Ali et al^152^ have examined 412 AA versus 217 CA PCa patients to link dysregulated gene expression and tumor aggressiveness in the AA population. They report the commonest 27 dysregulated genes including NKX3.1, APPL2, TPD52, LTC4S, ALDH1A3, AMD1, TPD52, and LTC4S were significantly upregulated, whose functions include response to oxidative stress, cell cycle regulation, cellular proliferation and apoptosis, migration, motility, cellular adhesion, and fatty acid synthesis and metabolism, as well as influencing pathways associated with oncogenesis, eg, constitutive PI3K/Akt and MAPK signaling.^152^ They conclude these deregulated signaling pathways in AA men may drive tumor aggressiveness and so account for PCa differences.^152^

Other studies have found both germline mutations in the EPHB2 gene,^153^ which encodes the EPHB2 receptor tyrosine kinase and BRCA gene,^154^ are linked to an increased incidence of PCa in AA men. Also, mitochondrial gene COI mutations found in 72.8% of AA men and 8.8% of CA men have been associated with aggressiveness in PCa disease.^155^ Of note, in a small cohort of AA patients, Lindquist et al^156^ found 17% of patients had a CDC27 to OAT gene fusion, the latter of which they note is influenced by AR signaling.^156^, ^157^, ^158^

Epigenetic differences affecting WNT signaling and stemness: Differing epigenetic changes between AA and CA men have been shown in a number of studies and have been concisely reviewed by Karakas et al.^96^ The genes RARβ2, SPARC, TIMP3, and NKX2‐5 are more highly methylated in AA compared to CA men, and NKX2‐5 and TIMP3 are hypermethylated, even in benign prostatic tissue of AA men.^159^ Devaney et al^160^ showed increased methylation of ABCG5 and SNRPN genes in AA versus CA samples, with reduced expression being associated with less aggressive disease in CA but not in AA PCa cell lines.^96^, ^160^ Tang et al^161^ found RARB gene methylation increased PCa risk in AA men, and another study in AA men found significantly differing methylation status in five ion‐binding genes (PRDM13, MAP2K5, ZFR2, MAST1, and CDH18) was associated with recurrent and aggressive disease.^162^ With regards to GSTP1 and CD44 methylation status between AA and CA men, there are conflicting reports. One study showed there was no difference,^163^ whereas Woodson et al^164^, ^165^ examining racial methylation status of GSTP1 and CD44, as well CDH1, ANXA2, RARb2, RASSF1, CAV1, and EDNRB, found it was increased in AA men. Moreover, GSTP1 methylation has been linked to a 13.3 × increased PCa risk in AA men compared to a 3.8 × increased risk in CA men.^8^, ^166^ Another interesting study looking at epigenetic differences between AA and CA men found tumors from AA men had hypermethylated loci at MC1, whereas CA men had hypomethylated loci at the MC3 cluster.^167^ The result of these epigenetic changes was reduced noncanonical Wnt signaling (Wnt/Ca+2 signaling) in AA men, and activation via the MC3 cluster for CA men. Furthermore, PI3K signaling and inflammatory pathways cause increased expression of MC1 genes.^167^ Looking at whole blood DNA from AA men, Moses‐Fynn et al^168^ assessed DNA methylation status in the genes RARβ2, TIMP3, SPARC, CDH13, HIN1, LINE1, CYB5R2, and DRD2. Overall, they found DNA promoter methylation was more common in AA men, and was linked to a number of clinical and pathological prognostic parameters including Gleason score.^168^

#### 3.2.5 Genetic variations and chromosomal instability

Differences in tumor biology resulting from pathways maintaining genomic stability as well as mutational burden between AA and CA men have been shown.^8^, ^96^, ^169^

Germline genetic mutations: Using linkage studies in hereditary PCa, HPC1 susceptibility locus on 1q24‐25 has been shown to increase risk of early onset and inherited disease, as well as having a higher prevalence in AA families with affected members.^170‐172^

### 3.3 Genetic polymorphisms

Genetic polymorphisms can be used as markers of genetic susceptibility as well as for disease prognosis. A study by Freedman et al,^171^ using whole‐genome admixture mapping analysis, identified a 3.8 mb region at 8q24 associated with PCa risk in AA men. It contained 9 genes, and a follow‐up study demonstrated seven SNPs conferring a higher risk of PCa to AA men over CA men.^96^, ^172^ One of these nine genes was the C‐MYC oncogene, discussed above, with the others listed in Table 2. Amundadottir et al^173^ found variants in the region of chromosome 8q24 (specifically allele 8 of microsatellite DG8S737) in 31% of normal AA men and 41% of AA men with PCa, compared to 13% and 19% in European groups, respectively. The population attributable risk was 16% in AA men compared to 8% in CA men, suggesting this allele may in part explain the increase incidence in AA men.^8^, ^173^ The link between PCa risk, chromosome locus 8q24, and AA ethnicity has also been confirmed in a number of other studies.^96^, ^174^, ^175^, ^176^, ^177^ A GWA study, in populations of AA and CA men of over 1000 each, showed four single‐nucleotide polymorphisms (SNPs) (rs2660753, rs13254738, rs10090154, and rs2735839) were associated with prostate cancer aggressiveness in both groups, whereas three SNPs were linked to PSA levels, and two other SNPs linked to Gleason score and disease stage in AA men only.^8^, ^178^ Another linkage analysis study in AA families showed prostate cancer susceptibility loci at 12q24 and 2p16.^8^, ^179^ Differing genetic polymorphisms between AA and CA man have been found in CYP3A4 and CYP3A43, the enzyme products of which play a key role in testosterone metabolism, as well as in the exon encoding amino‐terminal transcriptional domain of androgen receptor (discussed above).

**TABLE 2:** Nine Genes in 3.8 Mb region of 8q24 conferring higher risk to AA men^171^

GENE	Molecular changes	Cellular changes
C‐MYC	Upregulation of genes relating to transcription factors, mitochondrial biogenesis, RNA and protein biosynthesis, glycolysis	Cellular Proliferation with metabolic transformation and increased metastatic capability
MTSS1	Protein MTSS1 involved in actin and signaling receptor binding	Metastasis suppression
SQLE	Squalene Monooxygenase enzyme	Cholesterol biosynthesis and cellular proliferation
ZNF572	Zinc finger protein 572	Transcriptional regulation
C8orf36	Chromosome 8 open reading frame 36 protein	Zinc ion binding relating to double‐strand DNA break repair via homologous recombination
KIAA196 (also known as WASHC5)	WASH complex subunit 5	Regulation of actin assembly
TRIB1	Tribbles homolog 1	Protein degradation across a wide range of biological processes
FAM84B (also known as LRATD2)	Protein LRATD2	Cellular proliferation
TMEM75	Putative transmembrane protein 75	Embryonic cellular development

*Note*: Molecular and cellular changes taken from www.uniprot.org/, accessed 7/7/20. Variants in 8q24 (specifically allele 8 of microsatellite DG8S737) may be found in 30% of AA men, 41% of AA men with PCa, and confer a population attributable risk for PCa of 16%.^173^

DNA repair pathways: Studies have demonstrated DNA repair gene mutations and deletions in treatment naïve PCa,^34^, ^180^ in approximately 19% of localized and locally advanced disease cases after prostatectomy^58^, ^180^, ^181^ and in up to 20% to 30% of castration‐resistant disease patients.^34^ This includes genes involved in all aspects of DNA repair. Tonon et al^182^ examined genomic mutations in high‐risk, localized PCa for AA versus CA men. After an integrated genomic study, they demonstrated mutations (including deletions and rearrangements) at DNA repair genes PARP1, CDK12, and the RNA gene PVT1 for the well‐described PCa susceptibility locus 8q24.^182^ There is also an association between AR transcriptional activity and genomic instability,^180^ and AR signaling has been shown to regulate DDR.^183^ One of the most prevalent somatic mutations in the AR is Thr877Ala, which has discussed in the AR hypermutation section above, but how AR signaling influences genomic instability is not fully understood.

Castration‐resistant PCa (CRPC) has increased deficiency of DNA repair associated genes, including BRCA1, RAD54L, and RMI2. Interestingly, Li et al^184^ found the expression of these genes is suppressed by androgen‐receptor inhibitor enzalutamide, thus creating reduced homologous DNA repair ability and “BRCAness” in CRPC cells. Applying this therapeutically, they showed enzalutamide followed by the combination of enzalutamide and olaparib (a PARP inhibitor), induced cell death caused by DNA damage, and prevented clonal PCa cell proliferation.^184^

In fact, the study of DNA damage and repair response (DDR) pathways may be a source of other novel drugs, formed from hundreds of genes that produce pathways maintaining stability of the genome, ensuring a cell's long‐term survival. They can be classified into signaling pathways that repair single‐strand lesions (mismatch or base and nucleotide excision) and double‐strand lesions (homologous recombination (HR) or nonhomologous end joining (NHEJ)). Inactivation of core HR genes (eg, BRCA1/2) or HR associated genes (PTEN, CHEK2, SPOP, CHD1) and impairment of NHEJ pathways by BCL2 overexpression and TMPRSS2‐ERG fusion have been well described in PCa,^185^ and PARP inhibition can result in synthetic lethality by preventing not only base excision repair but alternative end joining pathways when both HR and NHEJ pathways are compromised. Recently, Yadav et al^186^ examined somatic mutations in the DNA repairome of AAs and CAs, using ultrahigh depth exome sequencing from 63 tumors. They found AA men had a higher rate of somatic mutations in DDR genes overall, and in their study, specific disparities in XPC (NER), ATR (MMR), and MBD4 (MMR) in the AA cohort. With this in mind, it is anticipated that AA men would respond well to check point therapies, and this should be a focus of future clinical trials.

Tumor mutational burden: Jaratlerdsiri et al^187^ performed whole‐genome sequencing in a direct study‐matched comparison of treatment naïve high‐risk prostate tumors between African and Caucasian men. They found a 1.8‐fold increase in AA men in smaller somatic mutations, rising to four‐fold when compared with published data (although this published data may have been from less high‐risk populations). These mutations were in oncogenic drivers, with approximately 30% of genes being novel and 79 % recurrent and associated with early oncogenesis. In keeping with previous studies, ERG fusions, PIK3, and PTEN loss were less in AA men, but they identified mutations in genes regulating calcium ion‐ATPase signal transduction.^187^

#### 3.3.1 Metabolic reprogramming

Mitochondrial factors: Recently, Xiao et al^188^ reviewed potential mitochondrial factors that might underlie racial disparity in prostate cancer, noting the considerable research that has linked mitochondrial health to incidence and disease aggression. For example, mitochondrial DNA G10398A polymorphism has been linked to aggressive prostate PCa in AA men,^189^ and other mitochondrial DNA mutations have been associated with increased tumorigenicity in mice experiments.^190^ Another study had found reduced mitochondrial DNA content correlates with adverse outcomes in AA patients.^191^ Finally, Chaudhary et al^192^ found reduced apoptosis related to mitochondrial dysfunction and defective heat shock proteins in AA men with prostate cancer, more specifically related to differences in mitochondrial unfolded protein stress response.^192^ More studies are required to further investigate these differences and potentially develop actionable ideas of clinical benefit related to them.

#### 3.3.2 Tumor microenvironment, Pro‐tumor inflammation, Escaping antitumor immune response, and Tumor‐induced angiogenesis

In comparison to Asian and Caucasian populations, those of African descent have rarer allele frequencies and more diversity in nucleotide composition. This results in differences in immune landscape that were adapted to their ancestral origins^193^ that have been shown to be specific to tissue types, but not specific cancers.^194^ The influence of these differences on immune surveillance, and the innate and adaptive immune responses, has been suggested to affect not only oncogenesis but also response to treatment and cancer outcomes.

Tumor microenvironment, inflammatory axis, and cytokines: The importance of the tumor microenvironment (TME) in PCa oncogenesis and disease progression has been established but few studies have examined its association with racial disparity.^15^ Blood vessels, adipocytes, and immune cells secrete various factors including growth factors and cytokines that constitute a unique landscape for PCa cells to proliferate and spread.^130^ Previous studies have shown altered tumor gene expression in AA men, with an increase in immune and inflammatory pathway activity, as well as cytokine signaling.^195^ In a recent study, Gillard et al^15^ investigated the function of fibroblasts isolated from PCa tissue in both AA and CA men and demonstrated a number of characteristics unique to the AA derived cells. Fibroblasts from AA men had an elevated growth response to testosterone, FGF2, and platelet‐derived growth factor, and caused increased proliferation and motility of PCa cells, as well as higher levels of myofibroblast‐activating markers, aSMA, vimentin, and tenascin‐C. A PCa cell line derived from AA men called “E006AA” had increased tumorgenicity in the presence of fibroblasts from AA men, and proinflammatory paracrine mediators (BDNF, CHI3L1, DPPIV, FGF7, IL18BP, IL6, and VEGF) were all increased in AA derived fibroblasts. Interestingly, a TrkB‐specific antagonist was able to reverse the protumorigenic effects of AA derived fibroblasts on the E006AA PCa cell line.^15^ They concluded the different characteristics of AA derived fibroblasts and its effect on the TME, may be a critical factor underlying PCa racial disparities. They also found an increase in collagen deposition and myofibroblasts as part of a “reactive stroma” and enhanced tenascin‐C protein in the extracellular matrix (ECM), all of which favor tumor growth and invasion.^15^

Kumar et al^130^ reviewing the immunobiological aspects of PCa racial disparities, highlighted elevated IL‐6 levels and the pathways it activates, including signal transducer and activator of transcription (STAT), mitogen‐activated kinases (MAPK), and phosphatidylinositol 3‐kinase (PI3K). The list of cytokines that may influence PCa racial disparities includes 14 interleukins, GM‐CSF, interferons, as well as TGF‐beta and TNF‐alpha. How these cytokines play out in the TME and in further stages of PCa invasion and metastasis and whether this differs in different ethnic populations require further study.

A multi‐institutional retrospective comparative genomic analysis led by our group on a large cohort of African American and European who underwent surgery demonstrates that tumors from African‐American men are enriched for immune‐related genes and suggests they could be better candidates for immunotherapy (manuscript under revision).

Epithelial to mesenchymal transformation: Epithelial‐mesenchymal transition (EMT) is a slow and prolonged change that occurs during embryonic development through oncogenesis. Epithelial cells gradually develop mesenchymal features, and there is increasing evidence that EMT may be linked to PCa metastatic progression as well as stemness and drug resistance.^196^ Investigating EMT differences, which may underlie racial disparity, Burton et al^197^ used Snail protein and Cat L activity as markers for mesenchymal transition. They found increased expression of mesenchymal protein (Snail, Vimentin, Cat L) and increased Cat L activity in PCa cells from AA men compared to normal and androgen‐dependent cells, and metastatic prostate cell lines in CA men.

Lymphocyte differences: A number of studies have examined tumor‐infiltrating lymphocytes in prostate cancer and their association with clinical and pathologic parameters.^198^ These found that extreme levels of lymphocytes, both high and low, are associated with a poorer prognosis, with the former being more frequent in published series.^198^ Flammiger et al,^199^ specifically investigating both T and B lymphocytes, found both very high and low levels of CD3+ T cells were associated with biochemical recurrence, but not B lymphocytes. Kaur et al^198^ further examining T cells subsets, but with a larger cohort including a large number of AA men, found higher FOX3P+ T cell density was linked to an increased risk of metastasis in AA men, albeit weakly, which requires validating with a larger cohort. Overall, T cell density was similar between different ethnicities, which Kaur et al^198^ found surprising given the differences in tumor inflammatory microenvironment as well as cytokine expression (which we have eluded to earlier in this review). They suggest this may be as a result of focusing on T cell populations alone.^198^

Copy number variations influencing the immune response: Differences in genome‐wide copy number variations (CNVs) between AA and CA men have been studied, revealing 27 chromosomal regions with significant copy number changes,^96^, ^200^ all of which were associated with altered gene expression. Using array‐comparative genomic hybridization in a larger cohort, four of these regions were validated and found to be significantly enriched with genes related to the immune response.^200^ Other differences in CNVs have been associated with differences in gene expression.^96^ More recently, in high‐risk AA families, Ledet et al^179^ identified a germline CNV at 14q32.33 encompassing the IGHG3 gene, which they propose may contribute to genetic susceptibility with an increased likelihood to higher incidence and mortality in the families studied, and potentially in the AA male population as a whole.

Racial differences in glucocorticoid pathway signaling influencing the immune landscape: In addition to AR signaling, glucocorticoid pathways are also known to influence immune status. AA men with low SES have been shown to have higher cortisol levels, and cortisol can influence transcription of a number of genes and, for example, NK and CTL function by causing promoter region histone acetylation for granzyme B and perforin genes.^201^

#### 3.3.3 Noncoding and micro RNA disparities

The evolution of enormous high‐throughput sequencing data has revealed the presence of a vast majority of transcripts that do not encode protein and are known as noncoding RNAs. Most of these noncoding transcripts have not been functionally characterized. The noncoding RNAs, depending on their size, are categorized either as short noncoding RNAs that include microRNAs (miRNAs), snoRNAs, transfer RNAs, etc., and the long noncoding RNAs that have a size greater than 200 nucleotides.

MicroRNAs (miRNAs or miRs) are a class of small noncoding RNAs, 19 to 25 nucleotides in length that play a critical role in the regulation of gene expression. They act by either degrading or inhibiting translation of target mRNAs.^202^ miRNAs have been demonstrated to play critical roles in cancer development and progression,^202^, ^203^, ^204^ and can act either as tumor promoter or suppressor.^203^, ^204^ Several recent studies have reported differentially expressed miRNAs in PCa across different races. Ten differentially expressed microRNAs have been reported in prostate tumors from AA and CA men and they form a balance with mRNAs to drive oncogenesis.^205^ Theodore et al showed a 2.2‐fold to 13.3‐fold increase in the expression of miR‐26a in AA PCa cell lines, when compared to CA cell lines.^206^ Recent studies in PCa have also shown association of differentially expressed microRNAs with AR and AR V7 expression in AA versus CA men.^207^, ^208^ Interestingly, a recent study also reported polymorphisms of miR‐196a2 rs11614913, miR‐146a rs2910164, and miR‐499 rs3746444 as risk factor for developing PCa in Asian people.^209^

Long noncoding RNAs are implicated in both physiological and pathological states, and several lncRNAs have been reported to regulate cancer development and progression. Research of lncRNAs in PCa has revealed its role in cancer development and progression, epigenetic regulation (eg, *CTBP1‐AS*, *HOTTIP*, *NEAT1)* ^210^, ^211^, ^212^, transcriptional regulation (NEAT1, SChLAP1),^210^, ^213^ as decoys and also as a sponge for microRNA (eg, *PTENP1*, *KRAS1P*, and *PCAT1*).^214^, ^215^, ^216^, ^217^, ^218^

Studies have also found that, in prostate cancer, about 50% of disparities are seen at the 8q24 locus.^174^ The long noncoding RNA, plasmacytoma variant translocation 1 (PVT1) at 8q24.21 plays an oncogenic role in prostate cancer,^219^ and has been implicated in prostate cancer invasion and metastasis.^220^ Tonon et al in a recent study reported higher expression of PVT1 in tumors from African Caribbean men compared to French Caucasians.^182^

These studies demonstrate the relevance of exploring the expression and function of noncomplex molecular regulatory networks with coding genes, and may help develop better prognostic and therapeutic markers for prostate cancer patients of different ethnicity.

## 4 ANATOMICAL AND MOLECULAR CORRELATIONS

Another recent study explored potential anatomical differences and molecular correlations in PCa between AA and CA men.^221^ In a retrospective cohort of close to 300 men, they confirmed previous findings that ETS positive tumors (with either ERG or other ETS transcription factor fusions) were commoner in CA men, whereas SPINK overexpression was more common in AA men. In keeping with the studies of Sundi et al,^222^ they found in cases defined as low risk preoperatively, anterior location was more common in AA men than CA men (50% vs 20% respectively).^221^ However, racial differences in molecular subtype did not persist when tumors were analyzed by location, and anterior tumors had higher volume, lower PSA density, and higher risk‐genomic classifier scores, suggesting a possible propensity to increased disease progression in the future.^221^

## 5 DIFFERENCES FOR AA MEN IN PUTATIVE DISEASE SUBCLASSES

Khani et al,^223^ examining radical prostatectomy specimens in over 100 AA men and CA men, allowed a direct comparison of some of the genomic classifications mentioned above. Specifically, they compared ERG rearrangement, SPINK1 overexpression, PTEN deletion, and SPOP mutation, and found, for AA men, ERG rearrangements were significantly less (27.6% vs 42.5%), SPINK1 overexpression was significantly higher (23.4% vs 8.2%), and reductions in PTEN deletion and SPOP mutations approached significance (6.9% vs 19.8% and 4.5% vs 10.3%, respectively).

## 6 ACTIONABLE IDEAS AND NOVEL IMMUNOTHERAPIES

### 6.1 Prevention

Apart from age, race, and family history, risk factors for PCa are poorly described.^9^, ^224^ Epidemiological studies looking at various factors, such as housing, job discrimination, and economic issues, resulting in certain dietary, lifestyle, and social behaviors have tended to focus on Caucasian populations,^9^ without demonstrating any underlying specific causative agents.

Powell et al,^225^ however, have demonstrated genomic abnormalities in prostate cancer with links to obesity, hypertension, and diabetes in AA men. They used microarray methods to assess RNA expression levels of 517 genes known to be linked with PCa from AA and CA men. They listed 22 genes in AA men with the most significantly differential RNA expression, providing insight into the intersection of prostate cancer with dietary and social behaviors in AA men that may result from underlying socioeconomic issues. IL6, IL8, IL1B, CXCR4, and FASN showed significantly higher levels,^225^ and since FASN polymorphisms have been associated with elevated BMIs, they suggested FASN maybe a genetic biomarker link between obesity and poor PCa outcome. Moreover, FASN inhibition may have therapeutic role, especially in obese AA men.^226^ Elevated expression of cytokines also links to obesity, hypertension, and metabolic syndrome, all of which have increased incidence in AA men. Clinically, this emphasizes the benefits of maintaining a healthy lifestyle and lends weight to public health drives in AA men to lower their incidence of PCa.^225^ IL6 specifically correlates with high‐risk Gleason scores and AR signaling (via STAT3, MAPK kinase, and PI‐3 kinase/AKT pathways),^225^, ^227^, ^228^, ^229^ and PI‐3 Kinase/AKT pathway signaling is also upregulated in diabetes, obesity, and hypertension,^230^ which itself can upregulate AR signaling in PCa.^225^, ^231^, ^232^ Moreover, Mitin et al^233^ have found diabetes mellitus was significantly linked to aggressive cancers in a cohort of men undergoing radiotherapy treatment, independent of ethnicity. Since hypertension is commoner in AA men, and AKT is activated in hypertensive men by angiotensin II, Powell et al^225^ suggest this supports the notion that the prostate cancer AKT signaling is the highest in hypertensive AA men.

When epidemiological studies have involved AA men, it is clear that low socioeconomic status increases risk of high‐grade PCa,^9^, ^234^, ^235^, ^236^ and since it is not clear how socioeconomic and environmental factors interact with AA genomic susceptibility, targeted “preventative” measures are not yet available. Notwithstanding the results of Powell et al^225^ above, this means that current activities directed at AA men are public health measures, education, and opportunistic screening with a view to redressing some of the social, cultural, and healthcare access issues. As discussed earlier, two recent epidemiological studies have demonstrated that when AA men have equal access to diagnosis and treatment, disparities in outcomes no longer exist.^4^, ^5^

With this in mind, we suggest a number of specific initiatives and “Paths to Victory” to improve early diagnosis and potentially reduce incidence and mortality of prostate cancer in AA men. This includes addressing lifestyle, behavioral, and access to healthcare problems and focusing on comorbidities for AA men and inflammatory etiological associations as they relate to disparate prostate cancer outcomes. For the former, initiatives may comprise of AA specific prostate cancer clinics, race‐specific prognostic tools for AA men entering active surveillance programs (see Biomarker section below), exercise, smoking cessation, and diet interventions, and developing AA specific MRI imaging protocols. With regards to managing comorbidities and associated inflammation that may contribute to etiology, we propose careful diabetic control for those AA men affected, reducing obesity with associated metabolic syndrome, and developing mind and body intervention programs (potentially in a trial setting). All of these initiatives could potentially be orchestrated in the setting of an AA specific prostate cancer facility mentioned above.

Once screened for hereditary disease, the prospect of preclinical gene therapy to prevent prostate cancer from developing is an exciting one. Gene therapy remains in its infancy, and currently clinical trials aimed at preventing the onset of disease are directed at younger individuals with such conditions as muscular dystrophy, hemophilia, and inherited metabolic disorders. The results are encouraging, but there are no studies examining specifically hereditary prostate cancer.^237^, ^238^

### 6.2 Clinical trials involving AA men

Overall, the recruitment of AA men for prostate cancer trials has traditionally been a challenge, and disappointingly, the statistics for AA men's involvement in PCa trials is not improving. A recent study showed the % of AA men has reduced from 11.3% in 1995 to 2.8% in 2014,^239^ and overall, reviewing 51 trials from 1987, 96% of those recruited were non‐Hispanic White men. In part, the reasons for this are the same as those suggested for the differences in incidence and mortality, such as lifestyle issues and healthcare access problems, and Recsok et al suggest other reasons are negative beliefs about trials, and lack of knowledge about what is involved in taking part.^239^ With these figures in mind, there is an urgent need to plan novel recruitment techniques specifically targeting AA patients, in order that they are fairly represented in future trials.

## 7 SPECIFIC BIOMARKERS FOR THE AA POPULATION

Biomarkers for PCa have the potential to inform diagnosis and prognosis, as well as influence treatment decisions at all stages of the disease,^9^ especially when they correlate with aggressive phenotypes.^9^, ^240^, ^241^, ^242^, ^243^, ^244^ In a recent review, Rebbeck^9^ highlights markers for all stages of the disease across AA and CA men. Apart from TMPRSS2:ERG, which is only present in 20% of AA men versus 62% of CA men,^245^ most biomarkers considered were more prevalent in AA men, especially in the early stages of oncogenesis (eg, TMPRSS2/ERG translocation/fusions, telomere dysregulation, GSTM1 hypermethylation, and C‐MYC alteration), during the metastatic stage (eg, PTEN loss and telomerase activation) and for castration resistant disease (eg, AR mutation and activation) (Rebbeck, 2016‐9). For localized disease, the increase in prevalence of common biomarkers linked to specific genomic abnormalities was less marked (eg, loss of P27 and NXK3.1). As well as for TMPRSS2:ERG translocation status, other studies have highlighted biomarker differences between AA and CA men including SPINK1, AMCAR, NXK3.1 ERG, SRD5A2, Ki67 GOLM1, and AR.^223^, ^246^ SPINK1 overexpression was associated with higher risk disease,^223^ but the prognostic value of TMPRSS2:ERG translocation status is less well described.^9^ Nevertheless, TMPRSS2:ERG translocation status may have prognostic value when associated with other risk factors: for example, in the presence of obesity, it is linked to poor disease outcomes,^9^, ^247^ and Echevarria et al^248^ recently demonstrated 81 ETS dependent genes that were AA race specific and overexpressed in AA men with BCR, potentially providing a useful biomarker of more aggressive disease.

Some of the findings of Powell et al^225^ have been discussed with regards to links of genomic signatures of prostate cancer to obesity, hypertension, and diabetes. They also found increased RNA expression of CXCR4 and BMP2 in AA men. BMP2 is involved in metastasis to bone^249^ and CXCR4 is a chemokine receptor regulating metastatic behavior in PCa cells and associated with aggression and treatment resistance.^225^, ^250^ Both of these may have actionable roles as prognostic biomarkers associated with aggressive phenotypes.

Sanchez et al^251^ examined immunoseroproteomic profiling in AA men with PCa. They demonstrated differing autoantibody response to tumor cell ENO1 (one of the glycolytic enzymes), related to differing posttranslational modification of the enzyme itself. They suggest further proteomic analysis of antitumor antibody responses in AA men compared to CA men may help to unveil aggressive disease phenotypes and so guide treatment choices.

The Decipher Genomic Classifier is a 22 gene mRNA based prognostic tool used after RP to assess future risk of BCR, metastatic progression, and mortality. Its utility has recently been validated in a cohort of AA men with equal access to healthcare, and in fact was potentially found to perform better than in CA men, although a study with larger numbers is needed to confirm this.^252^

McCabe et al^253^ outline PTEN as a prognostic and predictive biomarker in prostate cancer. PCa patients will have PTEN mutations in 2% to 14% of cases and copy number loss in 12% to 41% of cases. Moreover, PTEN loss is more common in mCRPC and is a marker for reduced PFS.^253^

Potential specific biomarkers for AA men have been presented, of which some, for example, those linked to RNA sequencing, proteomic analysis and immune response gene expression may also be targets for novel immunotherapeutics. Clinicians treating patients with PCa will be very familiar with the currently available biomarkers (PSA, 4K test, PCA3, Select MDx, Confirm MDx, Oncotype MDx, Prolaris, and Decipher). Given the differences in tumor biology that have been described, the hope is that research will develop biomarkers and gene panels to provide prognostic information and aid in treatment decisions specifically for the AA population.

## 8 TREATMENT

Prostate cancer treatment has seen a number of advances in recent years and, in response to a deepening understanding of molecular pathways underlying the evolution of the disease, the concept of “Precision Medicine” has emerged.^254^ This aims to provide individualized treatment, potentially in combination, once a patient's prostate cancer has undergone genomic profiling, over and above other treatments that have gained widespread acceptance (eg, cytotoxic therapy with radium‐223 or cabazitaxel, anti‐AR signaling with enzalutamide or abiraterone, and antitumor immunotherapy with sipuleucel.^255^, ^256^, ^257^, ^258^, ^259^, ^260^ Performing genomics on patients' solid tumor tissue, after biopsy or surgical treatment, to identify abnormalities in AR signaling, DNA repair, PI3K, WNT, and cell cycle pathways, as well as examining for immune/tumor microenvironment response signatures, allows the integration of clinical and genomic data to choose appropriate therapeutic trials for individual patients.^254^ Furthermore, potential biomarkers described above from research on RNA sequencing, proteomic analysis, and immune response gene expression in AA men may also reveal targets for exciting novel immunotherapies, not only highlighting an individual patient's genomic risk profile, but also those who may develop therapeutic resistance, so earmarking them for specific combination therapies.

Currently, some of the commonest abnormalities found on genomic profiling relate to deficiencies in homologous recombination DNA repair (genes such as ATM, PALB2, BRCA1, BRCA2, CHEK2, and the FANC (Fanconi's anemia) genes) or in microsatellite instability resulting from deficiencies in mismatch repair genes (eg MLH1, PMS2, MSH2 and MSH6).^260^ These will be considered individually, although it is worth noting that, at the time of writing, only DDR and MSI‐H treatments are currently actionable.

### 8.1 Treatment for homologous recombination DNA (HRD) repair deficiencies

PARP inhibition: PARP functions to repair single‐strand DNA breaks. If there is no PARP function, cells convert single strand to double strand DNA breaks, which are dealt with by HRD repair. When PARP is inhibited, and HDR repair pathways are deficient, the chromosome becomes unstable, and cell death occurs due to overwhelming genomic damage.^260^ In the phase 3 PROFound trial assessing olaparib vs enzalutamide or abiraterone (having had standard ADT) for mCRPC, the PARP inhibitor gave a nearly 4 month progression‐free survival advantage.^261^ The patient cohort receiving olaparib had at least one deficiency in ATM, BRCA1, or BRCA2, and so in this study, the effect is limited to patients with DDR deficiency. Similarly, a prior recent phase 2 trial of the PARP inhibitor, Olaparib, not specific for AA men, but in 50 patients with castration resistant metastatic disease, reported encouraging results. All patients had received multiple treatments prior to the trail, were resistant to taxanes, and 22% had more than 50% reduction in PSA values. Of the 32% of patients' biomarker positive for HDR deficiencies (as in deficient of the above genes on profiling), 88% responded and clinical outcomes were significantly better than for biomarker‐negative patients.^262^ As far as we are aware, differential responses between AA biomarker positive and CA biomarker positive men have not been evaluated and require further study.

Checkpoint immunotherapy: Following the success of sipileucel‐T,^259^ there has been a focus on immunotherapy for prostate cancer,^260^ and specifically checkpoint inhibitors linked to PD1 and CTL4 pathways. However, a phase 1 trial for Nivolumab (antiPD1)^263^ and two phase 3 trials for Ipilimumab (anti‐CTL4)^264^, ^265^ failed to show any convincing treatment responses.^260^ Other trails with anti‐PD1s (eg Pembrolizumab) have shown some PSA and radiographic response,^260^, ^266^, ^267^ raising the possibility of a subset of patients who may respond. On the basis that some BRCA deficient tumors have responded to PD1 inhibition, trials are exploring its use in tumors biomarker positive for HDR and MMR abnormalities.^260^, ^268^ How this may direct treatment for populations of AA men will also require further investigation (see Table 1.).

### 8.2 Treatment for microsatellite instability‐high tumors (MSI‐high) and mismatch repair (MMR) gene deficiencies

Only approximately 5% of advanced prostate cancers have MSI‐high and MMR abnormalities,^260^ and trials have been targeting this population with PD1 inhibition (eg, Pembrolizumab) in lung, colorectal tumors, and malignant melanoma, where the mutational load is high.^269^, ^270^, ^271^ However, a recent trial of castration resistant metastatic PCa with MSI, using combined enzalutamide with pembrolizumab, showed complete PSA response in 3 of 10 patients, one of whom had combined MSI and PDL‐1 tumor expression.^260^, ^266^ Similarly, biallelic somatic CDK12 mutations have been found in nearly 7% of mCRPC, which were not only associated with immune cell infiltration and enhanced checkpoint protein expression, but also increased efficacy of pembrolizumab PD‐1 inhibitor treatment.^272^ Once again, more study is required to assess how these responses may differ in a population of AA men.

### 8.3 Other immunotherapeutic initiatives

The use of Sipuleucel‐T for patients with mCRPC has been well described but a recent subanalysis of data from the original PROCEED trial demonstrated an 0S advantage of 9.5 months for AA men over CA men for all patients, as well as nearly 21 months when comparing AA to CA men with PSAs under 30.^273^ More study is required to understand the immunogenetics underlying this difference, and potentially develop focused AA therapies to exploit it further.

As mentioned earlier, our group has been applying the integrative use of clinical and genomic data, to uncover individual patients who may have high risk disease (with high risk of biochemical relapse), in combination with an immune/TME response signature. Such patients have more CD8/3 T‐cells, lower AR‐receptor activity, more inflammation, and lower levels of immune suppressive inflammatory cells (T‐reg cells and MDSC cells). By identifying specific cohorts of AA and CA men in this way and offering them “Precision Medicine” with entry in targeted therapeutic trials, the differential response of AA men can be studied. The results of these investigations will be available in the future.

## 9 CONCLUSION

This review has outlined the molecular and genomic fundamentals of prostate cancer for all ethnicities, framed in Hanahan and Weinberg's updated “Hallmarks” of cancer.

Using this framework as a benchmark, the molecular and genomic differences for AA men have been described, which may in part explain the racial disparity in disease incidence, even though recent epidemiological studies have shown equal access for AA men results in equal outcomes.^4^, ^5^ Emphasizing genetic abnormalities in prostate cancer, recommendations have been made for genomic profiling to integrate clinical and genomic data for the purposes of diagnosis, prognosis, and treatment planning for “Precision Medicine”. Treatment options have also been discussed, with a concise description of recent work in AA specific populations, showing an immune response signature, and detailing a number of targeted therapies. A summary of the current clinical trials that are active or recruiting can be seen in Table 3 (see below). It is encouraging that many of these are exploring lifestyle and educational initiatives, as well as therapeutic interventions, but clearly there is much work to be done to reduce incidence and mortality in AA men, and equalize current racial disparities.

**TABLE 3:** Listing PCa Trials specifically aimed at AA men (as of time of writing)

	Study Title	Condition	Intervention	Status/NCT Number	Location
1.	Green tea, black tea, or water in treating patients with prostate cancer undergoing surgery	Prostate cancer	Green tea Placebo Dietary decaffeinated black tea	Active (not recruiting) NCT00685516	Los Angeles, California.
2.	Anxiety in Black Men with prostate cancer: Validation of the memorial anxiety scale for prostate cancer in a sample of Black Men	Prostate cancer	Questionnaires of quality of life	Active (not recruiting) NCT00581672	New York, New York
3.	Biobank for African American prostate cancer research in Florida	Prostate cancer	Questionnaires Saliva samples Tumor tissue	Recruiting NCT03232411	Tampa, Florida.
4.	Molecular mechanisms underlying prostate cancer disparities	Prostate cancer	Biopsy or prostatectomy tissue	Recruiting NCT02229565	Durham, North Carolina.
5.	Improving health literacy in African American prostate cancer patients	Prostate cancer	Educational supplement Standard practice education	Recruiting NCT03322891	Atlanta, Georgia.
6.	An epidemiological study of genetic risk factors for prostate cancer in African American and Caucasian males	Prostate cancer	N/A	Active (not recruiting) NCT00342771	Baltimore, Maryland.
7.	Absorption and metabolism of Lyophilized Black raspberry food products in men with prostate cancer undergoing surgery	Localized and advanced prostate cancer	Lyophilized black raspberry confection Laboratory biomarker analysis Dietary intervention With others	Active (not recruiting) NCT01823562	Columbus, Ohio
8.	Men moving forward: A lifestyle program for African American prostate cancer survivors	Prostate cancer	Guided Lifestyle Program Intervention	Recruiting NCT03971591	Milwaukee, Wisconsin
9.	Lifestyle intervention for the reduction of prostate cancer disparities among African Americans	Prostate cancer (patient and relatives)	Exercise intervention Informational intervention Interview With others	Recruiting NCT04215029	Houston, Texas
10.	Informed decision making intervention in screening for prostate cancer of predominantly African American participants in a Community Outreach Program	Prostate cancer	Educational Intervention Digital rectal examination Pre‐test administration With others	Recruiting NCT02419846	Cleveland, Ohio
11.	Avelumab Plus 2nd‐generation ADT in African American subjects with mCRPC	Metastatic castration resistant Prostate cancer	Avelumab 2nd generation ADT (abiraterone or enzalutamide)	Recruiting NCT03770455	New Orleans, Louisiana
12.	Apalutamide and Abiraterone acetate in African American and Caucasian men with metastatic castrate resistant prostate cancer	Prostate cancer	ARN‐509 Abiraterone Acetate Prednisone	Recruiting NCT03098836	New Orleans, Louisiana

## CONFLICT OF INTEREST

A.K. Tewari has served as a as a site‐PI on pharma/industry‐sponsored clinical trials from Kite Pharma, Lumicell Inc, Dendreon, and Oncovir Inc. A.K. Tewari has served as an unpaid consultant to Roivant Biosciences and advisor to Promaxo. He owns equity in Promaxo. Z. S. Dovey, S. S .Nair, and D. Chakravarty declare no conflicts.

## AUTHOR CONTRIBUTIONS

All authors had full access to the data in the study and take responsibility for the integrity of the data and the accuracy of the data analysis. *Conceptualization*, Z.S.D., S.S.N., D.C., A.K.T.; *Project Administration*, Z.S.D., S.S.N., D.C., A.K.T.; *Supervision*, Z.S.D., S.S.N., D.C., A.K.T.; *Writing‐Original Draft*, Z.S.D.; *Writing‐Review and Editing*, Z.S.D., S.S.N., D.C., A.K.T.

## ETHICAL STATEMENT

Not applicable.

## Data Availability

Data sharing is not applicable to this article as no new data were created or analyzed in this study.
